# In this issue of *Current Oncology*

**Published:** 2008-12

**Authors:** M. McLean

This issue of the journal sees a diverse group of manuscripts that takes us through to the end of 2008 and of our 15th year of publication—an appropriate time to thank all our sponsors and supporters for their contributions and our editorial board and support staff for their many hours of effort.

In the area of clinical practice, Dr. Zeina Nahleh and colleagues provide an interesting review and analysis of outcomes for neoadjuvant chemotherapy in locally advanced breast cancer—including the inflammatory form—and of subsequent disease-free and overall survival. Dr. Barbara Melosky and coauthors report their experience with the use of second-line erlotinib following failure of first-line platinum-based chemotherapy, suggesting that this epidermal growth factor receptor–tyrosine kinase receptor inhibitor may be an efficacious and well-tolerated option for some patients. From the National Cancer Institute of Canada comes a report of a large prospective study designed to test the benefits of physical activity on well-being and survival in patients with stages II and III colon cancer following adjuvant chemotherapy. From McGill, Dr. Hamdy Elhateer and colleagues describe their experience in the management of pituitary macroadenomas, reporting an impressive 100% control rate after a median of 2 years of follow-up. My personal thanks go to Dr. Susan Solymoss for her scholarly editorial review of current substantial changes in the management of thrombosis in cancer patients.

A new educational series—Drug Developments in Contemporary Oncology—is introduced in this issue, and we are grateful to Drs. Matthew Warr and Gordon Shore for their introductory manuscript on small-molecule Bcl-2 antagonists as targeted therapy. This series will run throughout 2009.

Finally, on behalf of the team, compliments of the season to all of our readership!

